# Prognostic value of programmed death ligand 1 (PD-L1) in glioblastoma: a systematic review, meta-analysis and validation based on dataset

**DOI:** 10.1080/21655979.2021.1996515

**Published:** 2021-12-13

**Authors:** Huan Wang, Youchao Xiao, Xingguang Ren, Dahai Wan

**Affiliations:** a Department of Neurosurgery, First Hospital of Shanxi Medical University, Taiyuan, China; b Department of Neurosurgery, General Hospital of Tisco, Taiyuan, China

**Keywords:** Glioblastoma, immunotherapy, isocitrate dehydrogenase, prognostic value, programmed death ligand 1

## Abstract

Excellent prognostic value of programmed death ligand 1 (PD-L1) is observed in patients with other cancers; however, the prognostic value of PD-L1 in glioblastoma (GBM) remains unclear. Therefore, this meta-analysis evaluated the prognostic value of PD-L1 in GBM. We performed a systematic search in databases to screen eligible articles. The hazard ratio (HR) and 95% confidence interval (95% CI) were extracted from included articles. This meta-analysis included 15 studies, and the forest plot indicated that increased PD-L1 expression was associated with poorer overall survival (OS) of GBM (HR, 1.16; 95% CI, 1.05–1.27; *P* = 0.002). Furthermore, stratified analysis confirmed that PD-L1 expression was associated with unfavorable OS at the protein level (HR, 1.30; 95% CI, 1.13–1.48; *P*< 0.001) and messenger ribonucleic acid (mRNA) level (HR, 1.05; 95% CI, 1.00–1.09; *P*= 0.041). The analysis of a dataset verified the prognostic value of PD-L1 and revealed an association between PD-L1 mRNA expression and the status of isocitrate dehydrogenase (IDH). In conclusion, increased PD-L1 expression predicts unfavorable OS in GBM and may be a promising prognostic biomarker of GBM.

## Introduction

Glioblastoma (GBM) is the most aggressive tumor in the central nervous system and has an extremely poor median overall survival (OS), ranging from 8 to 14 months [1]. A maximum safe range of tumor resection followed by chemoradiotherapy has become the standard of care for patients with GBM [2]. However, recurrence is almost inevitable owing to diffuse microscopic infiltration of tumor cells into the surrounding of the normal brain tissue. Therefore, a small percentage of patients can survive 2 years with mainstream treatment strategies, and only approximately 5% of patients survive 5 years after diagnosis [3].

The increased survival of patients with GBM in the past few decades is primarily owing to new biomarkers and treatments derived from biomarkers, such as isocitrate dehydrogenase (IDH), epidermal growth factor receptor (EGFR) and O-6-methylguanine-DNA methyltransferase (MGMT). These markers provide opportunities for developing novel treatment strategies and can accurately assess patient outcomes [4–6].

Immunotherapy is considered a new therapeutic strategy and has achieved satisfactory results in treating various solid tumors. It has been reported that the immune system influences the development of GBM, indicating the great potential of immunotherapy [7,8]. The programmed cell death-1 (PD-1)/programmed death ligand 1 (PD-L1) axis is a classic immune checkpoint of future immunotherapeutic strategies. Clinical trials of immunotherapy against the PD-1/PD-L1 axis combined with radiotherapy and chemotherapy for patients with GBM are ongoing. Owing to the good prognostic value of PD-L1, abnormally expressed PD-L1 has become a reliable prognostic biomarker for various solid tumors [9–12].

However, the value of PD-L1 expression in predicting the prognosis of GBM remains controversial. We hypothesized that high PD-L1 expression is a reliable prognostic biomarker associated with poor OS of patients with GBM. Therefore, we conducted a meta-analysis to comprehensively assess the prognostic significance of PD-L1 in patients with GBM.

## Materials and methods

This meta-analysis has been registered in the International Platform of Registered Systematic Review and Meta-Analysis Protocols under the registration number INPLASY202070079. The current systematic review and meta-analysis was conducted as per the Preferred Reporting Items for Systematic Reviews and Meta-Analysis (PRISMA) guidelines [13].

### Search strategy

We designed a search strategy focussing on minimizing bias and maximizing sensitivity. We performed a comprehensive literature search in four electronic databases (Embase, PubMed, The Cochrane Library and Web of Science) until 15 September 2021. We manually reviewed the reference lists of included articles and searched the OpenGrey database (www.opengrey.eu) to identify additional studies and gray literature. The search strategy is demonstrated in Supplementary Material 1.

### Study selection and quality assessment

The inclusion criteria were as follows: 1) a prospective or retrospective cohort study involving the prognostic value of PD-L1 expression in GBM and 2) articles directly providing the hazard ratio (HR) and 95% confidence interval (CI) or the Kaplan–Meier (K–M) curve from which we could extract the HR and 95% CI. The exclusion criteria included the following: 1) *in vitro* or animal experiments, 2) articles not written in English and 3) conference abstracts, reviews, correspondence, comments and case reports.

Selected studies were assessed using the modified Newcastle–Ottawa Scale (NOS) [14], which was used to evaluate the quality of nonrandomised studies. The scale was focussed on three categories as follows: 1) selection of participants, 2) comparability and 3) outcome. Furthermore, the total score ranged from 0 to 9 points, and studies with a score ≥6 were considered high quality. Two authors independently assessed each study; disagreements in ratings were resolved by consensus or through consultation with a third author.

### Data extraction

Two researchers independently extracted the data from selected studies, and a third reviewer adjudicated discrepancies. The extracted information from selected articles included the following: name of the first author, year of publication, country, type of patient, index and method of detecting PD-L1 expression, the cutoff of PD-L1 expression, sample size, number of patients in the cohort, the treatment received by patients, HR and 95% CI, source of HR and its calculation method. If an article did not provide HR directly, we adopted the K–M curve to estimate HR indirectly. Researchers extracted HR and 95% CI with the solution provided by Tierney [15]. When both univariate and multivariate analyses were performed, HR obtained from univariate analysis was used unless the study only reported HR obtained from multivariate analysis.

### Statistical analysis and evaluation of publication bias

All HRs and 95% CIs extracted from the included studies were analyzed with the Stata 15.1 software (Stata Corporation, College Station, TX, United States). The forest plot figures presented the pooled HR and its 95% CI. The Higgins I-squared (I^2^) inconsistency test estimated heterogeneity across studies [16]. A random‐effects model was accepted only when we observed high heterogeneity (I^2^ > 50% and *P*< 0.05) across studies. We performed subgroup analysis to explore the source of heterogeneity; otherwise, a fixed‐effects model was accepted. Publication bias was visually evaluated using funnel plots and quantified using the Egger’s and Begg’s tests [17], where *P*< 0.05 was considered statistically significant. We used the trim-and-fill method to assess the robustness of the meta-analysis results in the presence of publication bias [18,19].

### CGGA database validation

The Chinese Glioma Genome Atlas (CGGA, http://www.cgga.org.cn) database is a nonprofit database containing clinical and multilevel biological information of patients with glioma. We selected the mRNAseq_325 dataset for further analyses. The mRNAseq_325 dataset contained 85 primary GBM and 24 recurrent GBM samples. A total of 108 GBM samples with complete clinical and survival information were included. We matched each sample with its PD-L1 mRNA expression. Detailed information of the included GBM samples can be downloaded from the CGGA database.

We performed unpaired t-test to assess the relationship between PD-L1 mRNA expression and clinical features, including age, sex, the status of IDH and type of patients (primary/recurrent GBM). These 108 GBM samples were divided into high and low PD-L1 groups based on the median expression. The K–M curve and log-rank test were used to validate the different survival rates between the low and high PD-L1 mRNA expression groups. The data were analyzed and visualized using GraphPad Prism 8.1 (GraphPad Software, Inc., La Jolla, CA, USA).

## Results

The prognostic value of PD-L1 expression in GBM remains unclear; therefore, we conducted a meta-analysis to explore the relationship between PD-L1 expression and OS of GBM. We comprehensively included 15 studies and extracted HRs. Subsequently, we explored the clinical significance of PD-L1 expression and validated the prognostic value of PD-L1 mRNA expression based on a dataset.

### Search results

**Figure 1.**
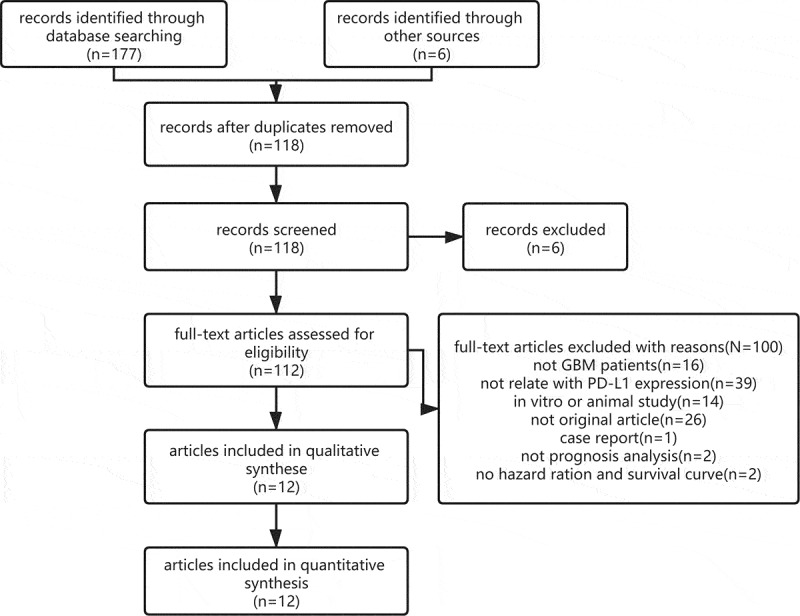
The flowchart of article selection

A flowchart of the study selection process is presented in Figure 1. A systematic search identified a total of 183 records, which are as follows: 48 from Embase, 44 from PubMed, 9 from Cochrane Library, 76 from Web of Science and 6 from other sources. After removing duplicates, 112 citations were retrieved for full-text examination; of which, 12 articles [20–31] were eligible and included in the meta-analysis. Two of the included articles [30,31] consisted of two cohorts. One of the included article [24] identified two indicators to assess the status of PD-L1 expression in the same cohort; therefore, these 12 included articles contained 15 studies. Each article has a NOS score of >6, indicating an excellent methodological quality (Table 1).

**Table 1.** Characteristics of the included studies

Ref	country	patient	index(assay)	cut off	total patients	treatment(method, No.)	PD-L1(±)	source of HR	HR(95%CI)	NOS
27	Denmark	GBM	protein(immunoflu-orescence)	percentage ≥10%	17	S + R, 2S + C, 15	6/11	K-M	1.50 (1.05–2.14)	7
30	Austria	GBM	protein(IHC)	percentage ≥5%	117	S + R + C, 102unknow,15	44/73	K-M	1.18 (0.75–1.87)	8
23	China	GBM	protein(IHC)	percentage ≥5%	62	NR	33/29	K-M	1.32 (0.96–1.82)	7
25	Korea	GBM	protein(IHC)	percentage ≥5%	54	S, 2S + R / S + C, 9S + R + C, 43	17/37	Cox regression	3.06 (1.16–8.06), UA	8
29	Korea	GBM	protein(IHC)	percentage ≥5%	115	S + R + C, 93S + R, 12S, 10	37/78	Cox regression	1.79 (1.10–2.91), UA	8
28	China	GBM	protein(IHC)	percentage ≥5%	20	S, 10S + C, 3S + R, 1S + R + C, 6	9/11	Cox regression	0.65 (0.25–1.69),MA	8
31	America	rGBM	protein(IHC)	percentage ≥5%	60	NR	22/38	Cox regression	1.96 (1.11–3.45), MA	6
21	China	GBM	protein(IHC)	score >4	47	NR	29/18	K-M	1.31 (1.00–1.72)	8
22	Denmark	GBM	protein(immunoflu-orescence)	expression leve ≥50%	163	NR	81/82	Cox regression	0.89(0.66–1.23), UA	7
30	Austria	GBM	mRNA(RNA-Seq)	expression level≥50%	446	NR	223/223	Cox regression	1.14 (0.96–1.37), UA	8
26	America	GBM	mRNA(Microarray)	expression level≥37%	152	NR	56/96	Cox regression	1.54 (1.05–2.28),UA	8
20	Japan	GBM	mRNA(RNA-Seq)	expression level≥50%	158	NR	79/79	K-M	1.07 (0.88–1.29)	7
31	America	rGBM	mRNA(RNA In Situ Hybridization)	expression leve ≥50%	60	NR	30/30	Cox regression	0.84 (0.50–1.41), MA	6
24	China	GBM	mRNA(RNA-Seq)	NR	152	NR	NR	Cox regression	1.08 (1.01–1.16), UA	6
24	China	GBM	mRNA(RNA-Seq)	NR	214	NR	NR	Cox regression	1.00 (0.94–1.07), UA	6

GBM, glioblastoma; rGBM, recurrent glioblastoma; Ref, reference; IHC, immunohistochemistry; RNA-seq, RNA sequencing data; NR, not report; MA, multivariate analysis; UA, univariate analysis; K–M, Kaplan–Meier curve; NOS, Newcastle–Ottawa Scale. S, surgery; R, radiotherapy; C, chemotherapy.

### Characteristics of the included studies

The main characteristics of the 15 included studies performed retrospectively and published from 2013 to 2021 are summarized in Table 1. Most studies were conducted in Asia (China, Korea and Japan). There were only two studies [31] that exclusively consisted of patients with recurrent GBM. The sample sizes of these 15 studies ranged from 17 to 446. Nine studies detected PD-L1 protein expression based on immunohistochemical (IHC) analysis or immunofluorescence. Six studies detected PD-L1 mRNA expression using microarray, mRNA-sequencing or RNA *in situ* hybridization assay. The cutoff value for determining the status of the PD-L1 was not reported in one article [24]. However, the definition of positive PD-L1 expression varied greatly among the included studies. HRs and 95% CIs were extracted directly based on Cox regression in 10 studies and from the K–M curve in other studies. In four studies [25,27,29,30], the patients were treated with surgery and chemoradiotherapy. However, no study recorded patients receiving immunotherapy.

### Association between PD-L1 expression and OS of GBM

**Figure 2.**
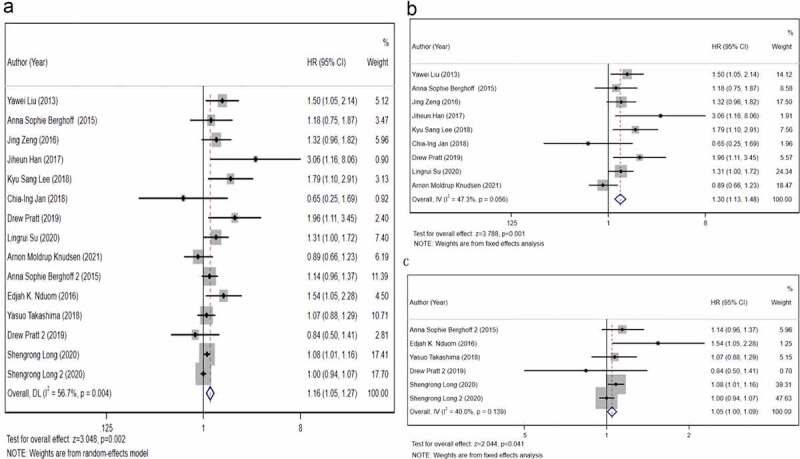
The primary meta-analysis. The relationship between overall survival (OS) and programmed death ligand 1 (PD-L1) expression. a) PD-L1 protein expression b) PD-L1 messenger ribonucleic acid (mRNA) expression c). The pooled hazard ratio (HR) of all included studies is 1.16 (95% CI, 1.11–1.46; *P*= 0.001); high heterogeneity is observed across studies (I^2^ = 56.7%, *P*= 0.002). After dividing all included studies into two subgroups based on detection index (protein and mRNA); no high heterogeneity is observed within the two subgroups. The results reveal that PD-L1 expression is associated with a poor OS of glioblastoma (GBM) at protein level (HR, 1.30; 95% CI, 1.13–1.48; *P*< 0.001) and mRNA level (HR, 1.05; 95% CI, 1.00–1.09; *P*= 0.041)

To analyze the prognostic value of PD-L1 protein expression in GBM, we included nine studies that have detected the PD-L1 protein expression and applied a fixed-effects model. The results (Figure 2(b)) revealed that positive PD-L1 expression was associated with an adverse OS of GBM (HR, 1.30; 95% CI, 1.13–1.48; *P*< 0.001). In addition, we performed a meta-analysis using a fixed-effects model to analyze the prognostic value of PD-L1 mRNA expression. The forest plot (Figure 2(c)) indicated that higher PD-L1 mRNA expression correlated with poorer OS (HR, 1.05; 95% CI, 1.00–1.09; *P*= 0.041). When we combined these 15 studies, the pooled HR (Figure 2(a)) indicated that high PD-L1 expression was associated with a poor prognosis (HR, 1.16; 95% CI, 1.05–1.27; *P*= 0.002). These results indicated high heterogeneity (I^2^ = 56.7%, *P*= 0.004).

### Subgroup analysis and publication bias

**Figure 3.**
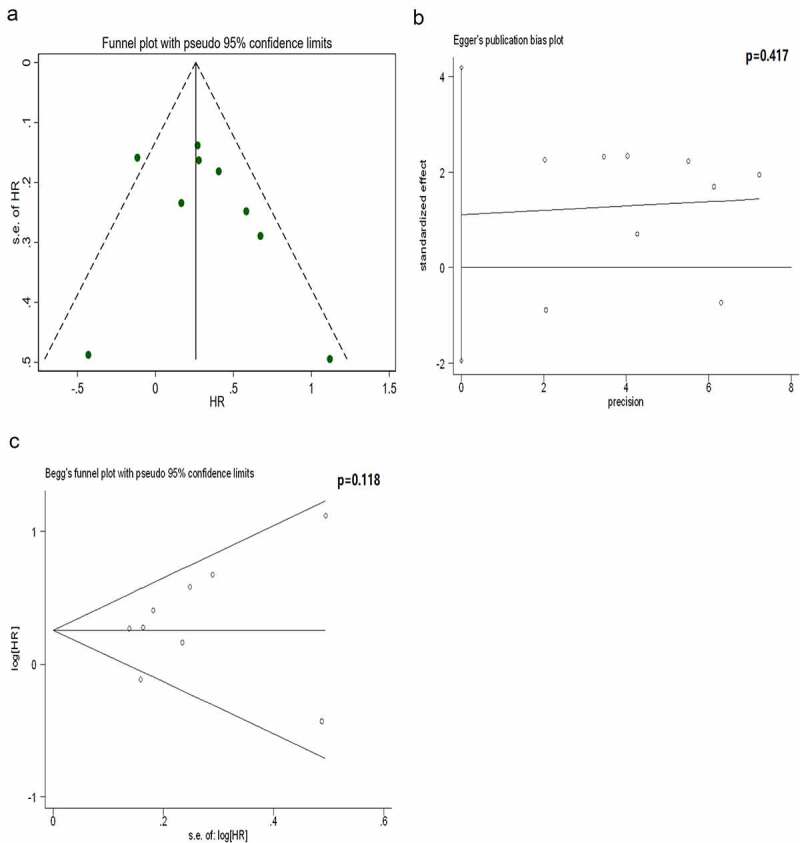
Analysis of publication bias for the protein subgroup. The results reveal that the funnel plot is symmetrical and the *P-*values of the Egger’s and Begg’s tests are 0.417 and 0.118, respectively. The symmetrical funnel plot, Egger’s test and Begg’s test reveal no publication bias within the protein subgroup

There was significant heterogeneity in our combined results (Figure 3(a)). Therefore, subgroup analysis was performed based on detection index, detection method, race, sample size, source of HR, cutoff and year of publication. Table 2 indicates that the heterogeneity was caused by five factors (heterogeneity between subgroups, *P*< 0.05), including detection index, detection method, source of HR, cutoff and year of publication; however, race (*P*= 0.51) and sample size (*P*= 0.083) did not influence heterogeneity.

**Table 2.** Subgroup analysis for the association between programmed death ligand 1 (PD-L1) expression and overall survival (OS)

Subgroup	Number of studies	Pooled HR(95%CI)	P value	Heterogeneity		Heterogeneity between groups (p value)
				I²	p value	
Detection index						0.003
protein	9	1.30 (1.13–1.48)	<0.001	0.473	0.056	
mRNA	6	1.05 (1.00–1.09)	0.041	0.4	0.139	
Detection method						0.007
immunofluorescence	2	1.12(0.88–1.41)	0.36	0.786	0.031	
immunohistochemistry	7	1.39(1.18–1.64)	<0.001	0.269	0.224	
RNA-sequencing	4	1.04(1.00–1.09)	0.045	0.172	0.305	
other	2	1.24(0.91–1.69)	0.176	0.703	0.066	
Race						0.51
asian	8	1.12 (1.01–1.25)	0.032	0.602	0.014	
caucasian	7	1.21 (1.00–1.46)	0.049	0.482	0.072	
Sample size						0.083
<100	7	1.34 (1.07–1.68)	0.012	0.415	0.114	
≥100	8	1.08 (1.00–1.18)	0.05	0.476	0.064	
Source of HR						0.038
Kaplan-Meier	5	1.21(1.07–1.38)	0.003	0	0.461	
Cox regression	10	1.05(1.01–1.12)	0.021	0.626	0.004	
Cutoff						0.001
protein staining percentage	7	1.45 (1.22–1.74)	<0.001	0.244	0.242	
mRNA expression level	4	1.13 (1.00–1.27)	0.043	0.255	0.259	
other	4	1.04 (0.99–1.09)	0.094	0.523	0.098	
Year of publication						0.006
<2019	9	1.22(1.10–1.35)	<0.001	0.376	0.118	
≥2019	6	1.04(1.00–1.09)	0.073	0.574	0.039	

Heterogeneity between the groups reveals that detection index, detection method, source of hazard ratio (HR), cutoff and year of publication are the sources of significant heterogeneity (*P* < 0.05); however, race and sample size are not (*P > *0.05).

Based on the Cochrane handbook, it is only appropriate to conduct publication bias assessment when there are no less than 10 eligible studies for the outcome measures [32]. Therefore, we only assessed the publication bias for the subgroup in which the protein was considered detection index, and this subgroup included nine studies. The results revealed that the funnel plot was symmetrically distributed. Both the Egger’s and Begg’s tests confirmed no publication bias in this subgroup (Figure 3(a-c)).

### Clinical significance and prognostic significance of PD-L1 mRNA expression in GBM

The characteristics of the CGGA-GBM cohort, which comprised 108 patients, are presented in Table 3. The median age and range, number of male patients, number of patients with primary GBM and number of patients with IDH-mutant GBM were 50 (11–79) years, 67 (62%), 85 (78.7%) and 19 (17.6%), respectively.

**Table 3.** Clinical features of the Chinese Glioma Genome Atlas-Glioblastoma (CGGA-GBM) dataset

Clinical characterics		number(percent)
age (median,range)		50 (11-79)
gender	male	67 (62%)
	female	41 (38%)
history ofrelapse	primary	85 (78.7%)
	recurrent	23 (21.3%)
status of IDH	wildtype	89 (82.4%)
	mutant	19 (17.6%)

IDH, isocitrate dehydrogenase.

**Figure 4.**
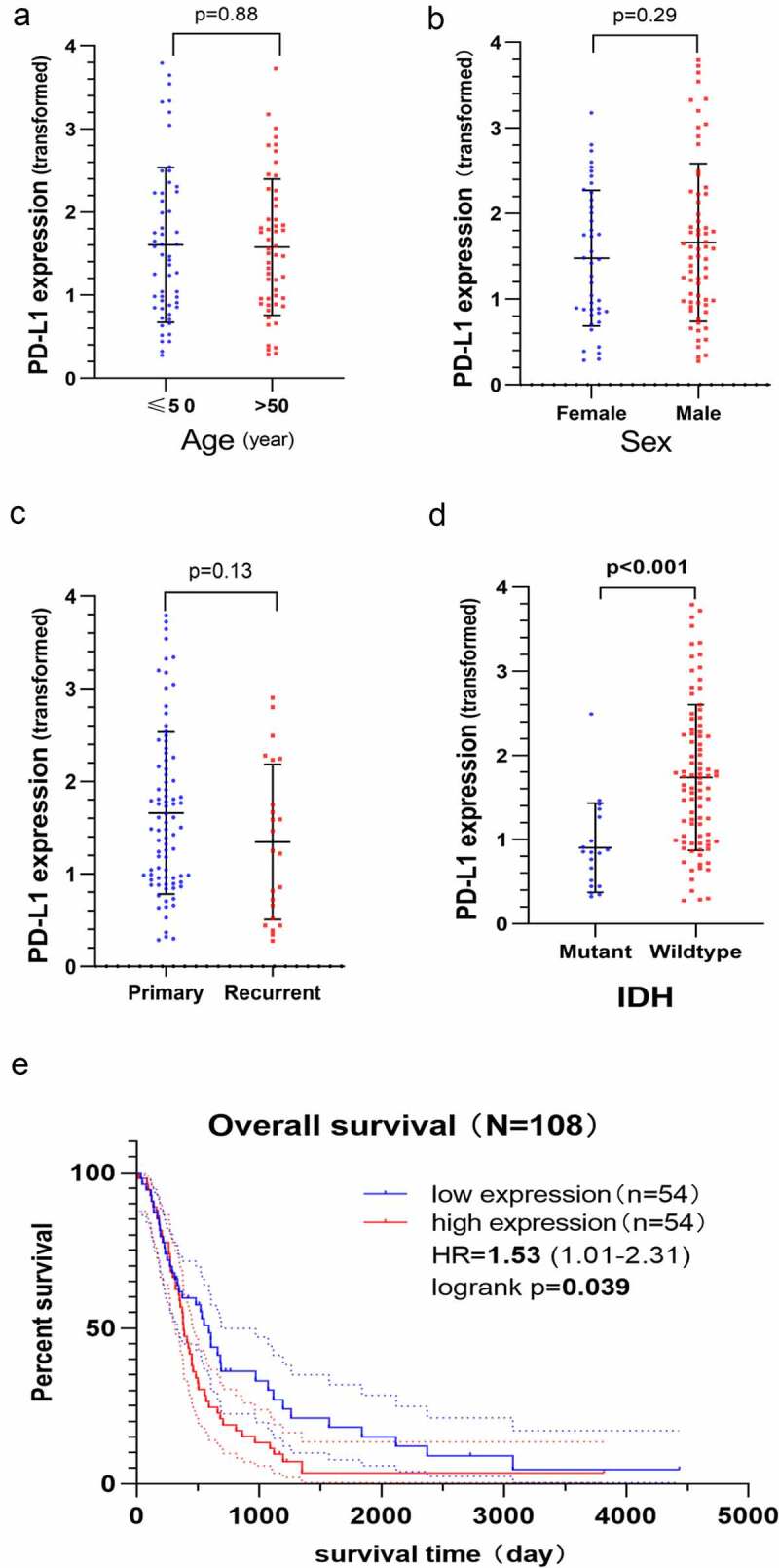
Clinical significance and prognostic value of PD-L1 mRNA expression based on the Chinese Glioma Genome Atlas-Glioblastoma (CGGA-GBM) dataset. The Y-axis represents the expression of PD-L1 mRNA transformed by log2 (1 + x) function. PD-L1 mRNA expression does not correlate with age a) sex b) history of relapse c) however, it correlates with the status of isocitrate dehydrogenase (IDH), and IDH wildtype GBM has higher PD-L1 mRNA expression level d). The Kaplan–Meier curve indicates that high PD-L1 mRNA expression is associated with a poor overall survival (OS) of GBM (HR, 1.53; *P*= 0.039) E)

The results revealed that PD-L1 mRNA expression was not different between older and younger patients (Figure 4(a), *p*= 0.88), male and female patients (Figure 4(b), *p*= 0.29) and patients with primary and recurrent GBM (Figure 4(c), *p*= 0.13). However, higher expression of PD-L1 mRNA was associated with patients with IDH-wildtype (Figure 4(d), *p*< 0.001). The K–M curve confirmed that higher PD-L1 mRNA expression predicted a poor OS (HR, 1.53; 95% CI, 1.01–2.31; *P*= 0.039, Figure 4(e)).

## Discussion

The blood–brain barrier (BBB) can affect material transport and cell migration and maintain the brain microenvironment’s homeostasis because of its unique selective permeability [33,34]. Consequently, the brain has long been considered an immune-free organ. However, some observed experimental phenomena seem to shatter this stereotype. GBM cells can secrete factors to break down the tight junctions forming the BBB, therefore, activated macrophages and lymphocytes can move across the BBB into the tumor tissue [35]. Brain tumor cells can cross the BBB and relocate in the peripheral blood [36]. Furthermore, emerging studies have supported that the immune microenvironment plays a crucial role in malignant growth and invasion, contributing to standard therapy resistance [37]. These observations confirm that the brain is not an isolated organ but closely communicates with the immune system. Destruction of the immunosuppressive microenvironment of GBM has become the focus of current immunotherapy strategies.

The anti-PD-1/PD-L1 axis immunotherapy has become one of the most critical immunotherapies for tumors. PD-L1 is an immune inhibitory receptor–ligand expressed on various cancer cells, and its receptor PD-1 is mainly expressed on T cells. Their binding inhibits the activation of T cells, protects the tumor from immune-mediated damage, helps tumor cells to survive and promotes immune escape [38]. Immune checkpoint inhibitors can block the binding of PD-1 and PD-L1, thus disrupting the formation of the immunosuppressive microenvironment. In non-small cell lung cancer and renal cell carcinoma, two PD-L1 inhibitors (durvalumab and atezolizumab) have demonstrated a manageable safety profile and great antitumour activity, including the prolongation of prognosis-free survival and inhibition of metastases [39,40]. However, anti-PD-1/PD-L1 monotherapy has demonstrated a limited efficacy in the treatment of GBM. Several clinical trials have revealed that anti-PD-L1 monotherapy did not prolong the survival of patients with GBM [41–44]. The low response rate essentially limits the potential efficacy of immunotherapy [45]. A study, however, suggested the possibility of combining PD-L1 inhibitors with radiation therapy for GBM based on the finding that PD-L1 inhibitors increased the sensitivity of GBM cells to radiation therapy [46]. Therefore, improving the response rate of patients with GBM to PD-L1 inhibitors and exploring the combination of anti-PD-L1 immunotherapy with other therapies has become significant challenges [47].

PD-L1, also known as B7-H1 or CD274, is a member of the B7-family [38]. PD-L1 regulates effective T cells function by binding to PD-1. The PD-L1/PD-1 axis may help tumor cells evade the immune system by suppressing the activation of various immune cells, including T cells, tumor-associated macrophages and natural killer cells [48]. PD-L1 expression and tumor mutational burden predict response to pembrolizumab in multiple tumor types. These biomarkers (alone/in combination) may help to identify patients who are more likely to respond to anti-PD-1 therapies across a broad spectrum of cancers [49]. A study on the PD-L1 inhibitor MPDL3280A observed the responses in patients whose tumor cells had high PD-L1 expression. However, PD-L1 expression in tumor-infiltrating lymphocytes predicted a more robust treatment response than PD-L1 expression in tumor cells [50]. High expression of PD-L1 in tumor-infiltrating lymphocytes is considered a good independent prognostic factor for patients with GBM (HR, 0.4; 95% CI, 0.2–0.8; *P* = 0.016), which is inconsistent with the prognostic significance of high expression of PD-L1 in GBM tumor cells [51].

GBM has a higher frequency of PD-L1 expression (30%–70%) than that of other solid tumors [52–55], such as melanoma, non-small cell lung cancer, prostate cancer and rectal cancer, suggesting that PD-L1 plays a strategic role in immunosuppression in various GBM molecular subtypes. The World Health Organization (WHO) classification of the central nervous system used molecular biology to define different glioma subtypes possessing distinct clinical outcomes [56,57]. Several molecular markers, such as IDH, EGFR, MGMT and phosphatase and tensin homologue, can affect the formulation and implementation of treatment strategies for patients with GBM and can well predict the prognosis of patients. According to IDH status, GBM is mainly divided into two subgroups, including IDH-mutant GBM (approximately 90% of cases) and IDH-wildtype GBM (approximately 10% of cases). Regardless of GBM or lower-grade gliomas, IDH-wildtype gliomas have a poorer prognosis than that of IDH-mutated gliomas [56–58]. In this study, we observed that the expression of PD-L1 was related to the status of IDH, and patients with IDH-wildtype GBM had higher PD-L1 mRNA expression (Figure 4(d), *p*< 0.05). Previous studies have demonstrated that at the membrane protein level, IDH-wildtype GBM has a higher PD-L1 positive expression rate than that of IDH-mutant GBM (90.1% versus 9.1%, respectively) [31]. High expression of PD-L1 mRNA and protein contributed to the formation of the immunosuppressive tumor microenvironment, which may account for the short survival of patients with IDH-wildtype GBM to a certain extent. Correlation analysis revealed a significant positive correlation between EGFR and PD‐L1 expression (r = 0.654, *P*< 0.05) [21]. There was no significant association between PD-L1 expression and MGMT; however, PD-L1 expression correlated positively with CD3 + T-cell infiltration (*P*< 0.05) [29]. These results suggested that the expression of PD-L1 has different biological functions in the malignancy or different biological characteristics in different subpopulations.

Some antitumour immunotherapies targeting the PD-1/PD-L1 axis are entering clinical trials, and some studies are exploring the feasibility of PD-L1 in prognostic assessment. To the best of our knowledge, a meta-analysis has indicated an association between high/positive PD-L1 expression and adverse prognosis of GBM [59]. However, its conclusion may not be reliable owing to a small number of included studies (n = 3). Recently, several relevant studies have reported contradicting conclusions to the previous meta-analysis. Therefore, it is necessary to include these new studies, improve retrieval strategies and reevaluate the prognostic significance of PD-L1 expression in GBM.

The present meta‐analysis observed a significant association between high/positive PD-L1 expression and poor prognosis of patients with GBM (HR, 1.16; 95% CI, 1.05–1.27; *P*= 0.001), and high heterogeneity (I^2^ = 56.7%, *P* = 0.004) was also observed. The subgroup analysis revealed that the detection index, detection method, source of HR, cutoff and year of publication could reduce significant heterogeneity. We divided 15 studies into protein and mRNA groups. The results revealed that high PD-L1 mRNA and protein expression were associated with an unfavorable OS of GBM (Figure 2(b,c)). The absence of intra-group heterogeneity (Figure 2(b,c)) and intra-group publication bias (Figure 3(a-c)) further supported the reliability of this conclusion. In the CGGA-GBM cohort, we observed that the patients with IDH-wildtype GBM had higher PD-L1 mRNA expression than that of patients with IDH-mutant GBM. Furthermore, this cohort study confirmed that high PD-L1 mRNA expression predicted a poorer prognosis.

### Limitation

This meta-analysis had several limitations, which are as follows: (a) In the 15 included studies, different assessment methods, cutoffs and sources of HR may have caused significant heterogeneity. Stratified analysis based on these factors did not eliminate all the intra-subgroup heterogeneities. Subgroup analysis suggested that heterogeneity among these studies had complex mechanisms and was challenging to solve, (b) The treatment received by patients may affect their survival. The 15 included studies were retrospective instead of prospective, and there was no unified treatment method and standard, which may have been the source of heterogeneity, (c) Different GBM subgroups have different molecular characteristics, genetic backgrounds and distinct prognoses. The expression of PD-L1 was intimately correlated with different molecular characteristics; for example, this study confirmed the association between PD-L1 and IDH. Therefore, further studies are required to analyze the correlation between PD-L1 and GBM subgroups with distinct molecular characteristics.

## Conclusion

In conclusion, the mRNA expression level of PD-L1 was not related to the age, sex and history of relapse of patients with GBM but was related to the status of IDH. Moreover, elevated PD-L1 protein and mRNA expression levels were associated with poorer OS of GBM. Owing to the limitations we mentioned, more large-sample sized and multicentre prospective studies are required to validate the prognostic value of PD-L1 expression in GBM, especially in GBM subpopulations with different molecular characteristics.

## Supplementary Material

Supplemental Material

## Data Availability

All data are provided in the manuscript and can be obtained from the corresponding author.

## Highlights

- Increased PD-L1 protein expression correlates with an adverse OS of GBM.
- Increased PD-L1 mRNA expression correlates with a poor OS of GBM.
- PD-L1 mRNA expression is higher in IDH-wildtype GBM than in IDH-mutant GBM.
