# Supplementary material for: Prognostic value of programmed death ligand 1 (PD-L1) in glioblastoma: a systematic review, meta-analysis and validation based on dataset
Source: Bioengineered. 2021 Dec 13;12(2):10366–78. doi: 10.1080/21655979.2021.1996515 (PMC8809998; doi:10.1080/21655979.2021.1996515)
Supplement: Supplemental Material [file KBIE_A_1996515_SM1875.zip › Supplementary material 1 legend.docx]

**Supplementary material 1:** The details of the search strategy and results of each database.
